# METTL3 regulates WTAP protein homeostasis

**DOI:** 10.1038/s41419-018-0843-z

**Published:** 2018-07-23

**Authors:** Melissa Sorci, Zaira Ianniello, Sonia Cruciani, Simone Larivera, Lavinia Ceci Ginistrelli, Ernestina Capuano, Marcella Marchioni, Francesco Fazi, Alessandro Fatica

**Affiliations:** 1 Department of Biology and Biotechnology “C. Darwin”, Sapienza University of Rome, 00185 Rome, Italy; 2 Department of Anatomical, Histological, Forensic and Orthopaedic Sciences, Sapienza University of Rome, 00185 Rome, Italy; 3 Institute of Biology, Molecular Medicine and Nanobiotechnology, CNR, Sapienza University of Rome, Rome, Italy; 4 Istituto Pasteur Italia-Fondazione Cenci Bolognetti, 00185 Rome, Italy

## Abstract

The Wilms tumor 1 (WT1)-associated protein (WTAP) is upregulated in many tumors, including, acute myeloid leukemia (AML), where it plays an oncogenic role by interacting with different proteins involved in RNA processing and cell proliferation. In addition, WTAP is also a regulator of the nuclear complex required for the deposition of *N*^6^-methyladenosine (m6A) into mRNAs, containing the METTL3 methyltransferase. However, it is not clear if WTAP may have m6A-independent regulatory functions that might contribute to its oncogenic role. Here, we show that both knockdown and overexpression of METTL3 protein results in WTAP protein upregulation, indicating that METTL3 levels are critical for WTAP protein homeostasis. However, we show that WTAP upregulation is not sufficient to promote cell proliferation in the absence of a functional METTL3. Therein, these data indicate that the reported oncogenic function of WTAP is strictly connected to a functional m6A methylation complex.

## Introduction

*N*^6^-methyladenosine (m6A) is the most abundant internal chemical modification in eukaryotic mRNA and it can control any aspect of mRNA post-transcriptional regulation^1^. In mammals, the writer of m6A is a nuclear multicomponent complex composed of two *methyltransferase-like* proteins, METTL3 and METTL14, and the regulatory proteins *Wilms tumor 1-associated protein* (WTAP), *vir like m6A methyltransferase associated* (VIRMA, also known as KIAA1429), *RNA-binding motif protein 15* (RBM15) and *zinc finger CCCH-type containing 13* (ZC3H13)^1, 2^. METTL3 is the sole catalytic component of the complex while METTL14 functions in structural stabilization and RNA substrate recognition^3–5^. More recently, the human U6 snRNA m6A methyltransferase METTL16 has been shown to target intronic regions of pre-mRNAs and lncRNAs^6, 7^. Removal of m6A from transcripts occurs predominantly in the nucleus and requires the activity of the *alkB homologue 5 protein* (ALKBH5) and *fat mass and obesity-associated protein* (FTO)^1^. Several proteins, in both nucleus and cytoplasm, can read m6A modification and, eventually, regulate different phases of mRNA expression^8^. In particular, proteins containing the YTH domain were the first to be identified as m6A-specific “readers”. Notably, METTL3 itself can switch from writer to reader by moving in the cytoplasm where it can regulate the translation of specific mRNAs by direct binding to RNA and recruitment of eIF3^9, 10^.

The WTAP protein has been recently described as an oncogenic protein in different cancers, including acute myeloid leukemia (AML)^11^. WTAP was initially identified as an interactor of the Wilms Tumor-1 (WT-1) protein^12^, a zinc-finger protein that can act as both transcriptional and splicing regulator. Later on, WTAP was shown to form stable interactions with the Hakai protein (also known as CBLL1), a C3HC4-type RING finger containing E3 ubiquitin ligase whose expression is correlated to cell proliferation and tumorigenesis^13^, and different proteins involved in regulation of RNA processing and translation, including METTL3^14^. Interestingly, even if a large proportion of mRNAs associated with WTAP is also bound by METTL3, there are many mRNAs that are specifically associated with only one of the two proteins and upon knockdown only half of the misregulated genes are in common between the two factors^15^. Moreover, it has been proposed that WTAP form a stable complex with Virma, Hakai, Rbm15, and Zc3h13 (referred to as MACOM, *m*6A-METTL-*a*ssociated *com*plex) that acts beyond m6A methylation^2^. Thus, it is not clear if WTAP may have independent regulatory functions from the m6A modification complex that might contribute to its oncogenic role.

Here, we show that both the knockdown and overexpression of METTL3 protein results in WTAP upregulation, indicating that METTL3 levels are critical for WTAP protein homeostasis. In particular, we demonstrate that METTL3 levels may regulate WTAP expression at multiple levels by direct and indirect mechanisms that include mRNA translation and stability. However, we show that WTAP upregulation has on oncogenic effect only in the presence of a functional METTL3.

## Results and discussion

**Fig. 1:**
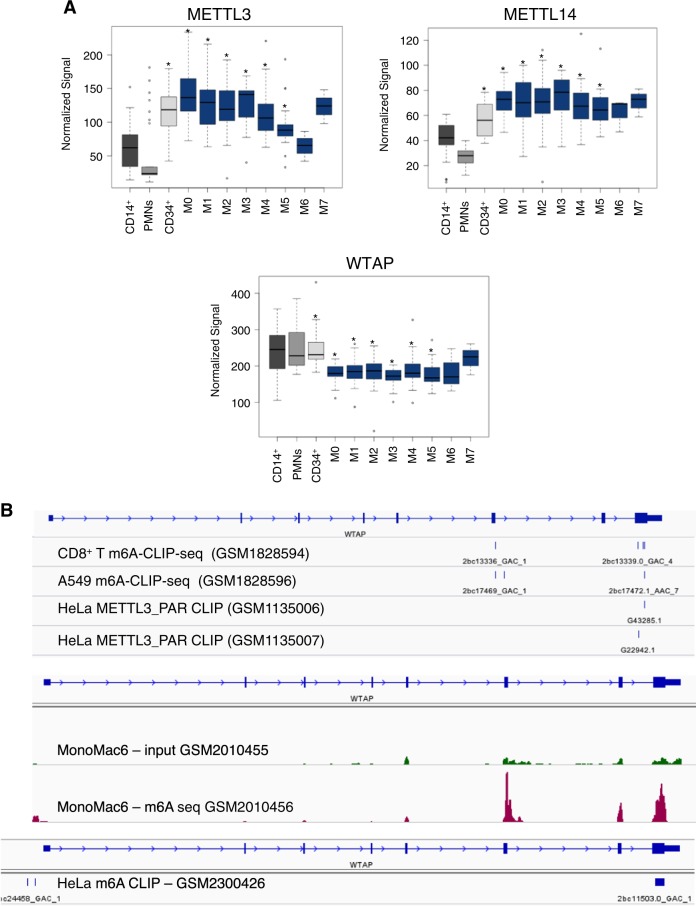
METTL3 and METTL14 are upregulated in AML. **a** METTL3, METTL14, and WTAP expression in AML of different FAB subtypes (in blue), M0 (*n* = 15), M1 (*n* = 43), M2 (*n* = 42), M3 (*n* = 17), M4 (*n* = 36), M5 (*n* = 22), M6 (*n* = 3), and M7 (*n* = 3); normal CD34+ hematopoietic progenitors (*n* = 22) and mature myeloid cells (in gray), normal CD14+ monocytes (*n* = 34) and polymorphonuclear leukocytes (PMNs, *n* = 30). Data were obtained from public microarray repositories. The box plots illustrate the distribution of expression values of the mean of all probes present in the microarray for indicated gene; the central solid line indicates the median; the limits of the box show the upper and lower percentiles. *p < 0.001 calculated on AML and CD34^+^ with respect to mature myeloid cells. Values for single probes are represented in Supplemental Figure S1. **b** Analysis of m6A peak and METTL3 binding in WTAP mRNA using published m6A CLIP (GSM1828594, GSM1828596, GSM2300426), METTL3 PAR-CLIP (GSM1135006 and GSM1135007) and m6A-seq (GSM2010455, GSM2010456) data, the bars in the m6A CLIP lane indicate m6A sites

To get insight into a potential role of the m6A methylation complex in AML, we first analyzed the expression of its components in a variety of AML subtypes, normal hematopoietic progenitor cells and mature myeloid cells (data from GEO and the Cancer Genome Atlas databases, TCGA). METTL3 and METTL14 mRNAs are significantly up-regulated in a high percentage of different AML FAB subtypes (the French–American–British classification of AML) compared to mature myeloid cells, while both genes are highly expressed in CD34+ progenitor cells (Fig. 1a and Supplemental Figure S1). The lack of significance in M6 and M7 AMLs is very likely due to the small number of samples. Moreover, it has been reported that both METTL3 and METTL14 are also highly expressed in AML compared to other cancers^16^. While our studies were in progress, different independent studies reported similar results showing a specific up-regulation of METTL3 and METTL14 in AML cells and a critical role for these proteins in AML cells survival and differentiation^17–20^. On the other hand, we observed low expression of WTAP mRNA despite the high levels of WTAP protein reported in AML^11^.

**Fig. 2:**
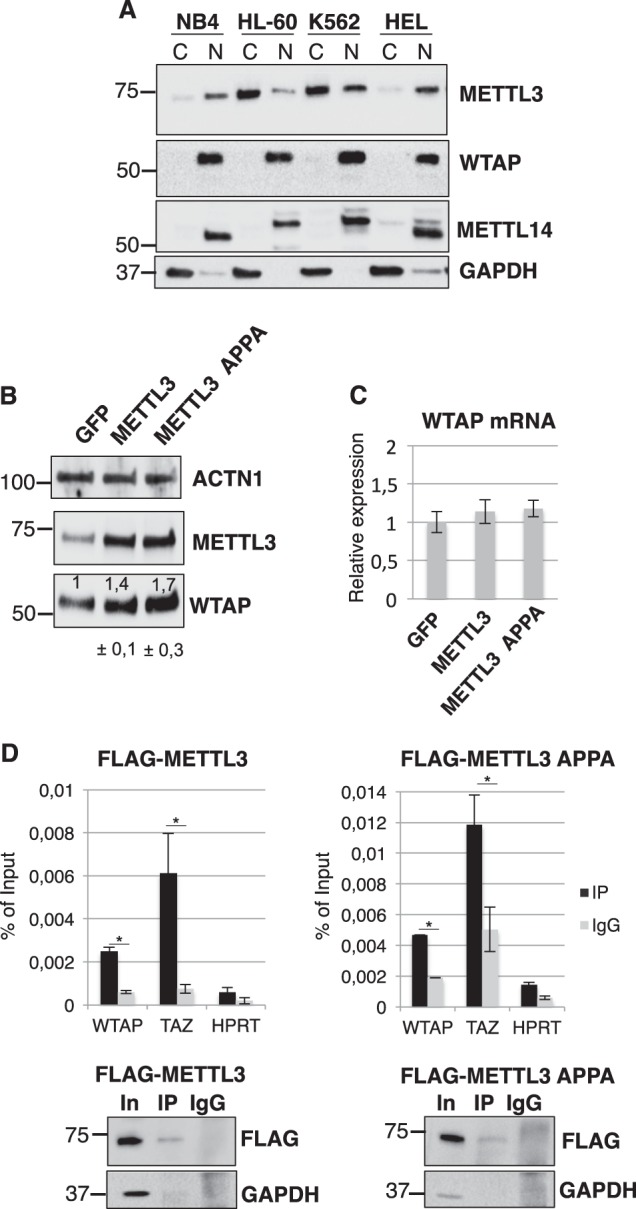
METTL3 is mislocalized to cytoplasm in AML and regulates WTAP expression. **a** Western blot analysis of METTL3 and METTL14 in nuclear, “N”, and cytoplasmic, “C”, fractions of different myeloid leukemia cell lines. WTAP and GAPDH were utilized as nuclear and cytoplasmic controls, respectively. **b** Western blot analysis of WTAP and METTL3 expression in K562 cells overexpressing METTL3 and METTL3 catalytic inactive mutant (METTL3 APPA). Densitometric analysis of WTAP/ACTN1 ratio from replicates (*N* = 5) is shown below with s.e.m. **c** qRT-PCR analysis of WTAP mRNA in the same cells. **d** Upper panel, qRT-PCR of CLIP experiments performed with FLAG METTL3 and FLAG-METTL3 APPA from cytoplasmic extract of K562 cells. Lower panel, representative Western blot analysis of FLAG-tagged proteins. Data are presented as ±SEM from three independent experiments. *p < 0.05

By analyzing published m6A-seq and m6A-CLIP experiments performed in different cell lines^17, 21–23^, including the AML cell line MonoMac6, we observed that WTAP mRNA is generally m6A methylated in exon 6 and in exon 8 (Fig. 1b). Furthermore, published PAR-CLIP data revealed binding of METTL3 within the m6A peak in the exon 8 of WTAP mRNA (Fig. 1b)^22^. We checked for METTL3, METTL14 and WTAP localization in several AML cell lines, and in all of them we detected METTL3 mislocalization in the cytoplasm while the other components of the m6A methylation complex were predominantly localized in the nuclear fraction (Fig. 2a). Therein, in view of these results, we hypothesized that METTL3 might contribute to the WTAP aberrant up-regulation observed in AML.

By using K562 leukemia cells stably expressing a doxycycline (dox) inducible METTL3 or METTL3 catalytic inactive mutant (aa395–398, DPPW → APPA, METTL3 APPA), we showed that METTL3 overexpression (48 h dox) resulted in increased levels of WTAP protein without concomitant increase in WTAP mRNA levels (Fig. 2b, c). Notably, METTL3 positively controls WTAP protein levels regardless its catalytic activity, similar to what has been shown for the translational control mediated by cytoplasmic METTL3 in lung cancer^9^.

Binding of METTL3 and METTL3 APPA to WTAP mRNA in cytoplasm of K562 cells was confirmed by CLIP experiments using stable cell lines carrying inducible FLAG-tagged expression cassettes (Fig. 2d). The METTL3 cytoplasmic mRNA target encoding for TAZ^9^ was utilized as positive control. Altogether these data showed that WTAP mRNA is m6A methylated and bound by cytoplasmic METTL3.

**Fig. 3:**
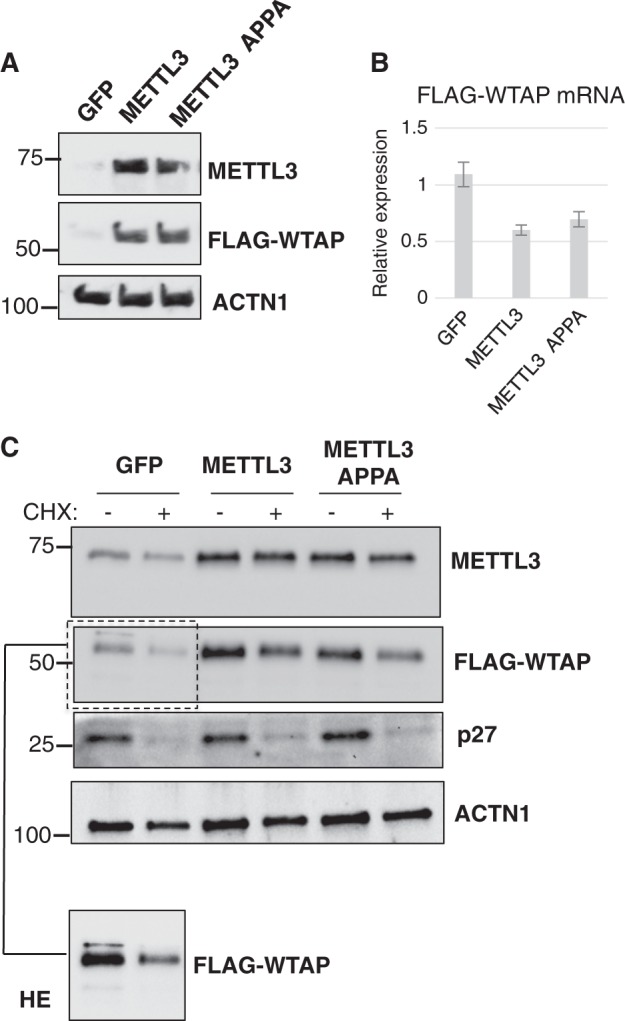
METTL3 regulates WTAP protein levels in HeLa. **a** Western blot analysis of HeLa cells transfected with a plasmid for transient expression of a FLAG-tagged WTAP together with plasmids expressing a GFP control, METTL3 or METTL3 APPA. **b** qRT-PCR analysis of FLAG-WTAP mRNA in the same cells. **c** Representative Western blot analysis of HeLa cells transfected with a plasmid for transient expression of a FLAG-tagged WTAP together with a plasmid expressing the wild type METTL3 protein, METTL3 APPA or control GFP and treated with cycloheximide for 4 h. Higher exposure (HE) of FLAG-WTAP in GFP transfected cells is shown. Data are presented as ±SEM from three independent experiments

To analyze if the observed phenotype was specific of AML cells, we transfected HeLa cells with a plasmid for transient expression of a FLAG-tagged WTAP construct containing only WTAP coding sequence together with plasmids expressing a GFP control, METTL3 or METTL3 APPA (Fig. 3a, b). Also in this case, we observed increased expression of FLAG-WTAP protein without concomitant increase of FLAG-WTAP mRNA expression.

It has been recently shown that METTL3 can bind m6A containing mRNAs^10^. We therefore examined whether m6A modifications in WTAP mRNA are necessary for METTL3 binding. We transfected a FLAG-tagged WTAP construct with a deletion in the region of exon 8 (FLAG-WTAP_Δ8), containing the m6A modifications, together with plasmids for METTL3 expression or control GFP (Supplementary Figure S2). The construct was already devoid of 5′- and 3′-UTR. Notably, we still observed increased WTAP protein levels and binding of METTL3 protein to WTAP mRNA. Moreover, binding of METTL3 and METTL3 APPA to FLAG-WRAP_Δ8 in cytoplasm of transfected cells was confirmed by CLIP experiments. These findings indicate that m6A modifications in WTAP mRNA are not required for METTL3 association.

In order to understand if the increase of WTAP protein depends on translation or protein stabilization, we performed overexpression of METTL3 protein in the presence of the translation elongation inhibitor cycloheximide (Fig. 3c). Thus, we co-transfected HeLa cells with the FLAG-tagged WTAP construct (see above) together with a plasmid expressing the wild type METTL3 protein, METTL3 APPA or control GFP. After 48 h from transfection we treated cells with cycloheximide for 4 h (+CHX). We used non-treated cells as control cells (−CHX). Properly occurred translational block was verified by checking the level of p27 protein, a well-known short half-life protein in proliferating cells^24^. In contrast, Actinin (ACTN1) protein levels were found to be constant throughout the experiment, thus it was utilized as endogenous control. Interestingly, we observed that after cycloheximide treatment WTAP protein decreased at the same levels in cells expressing METTL3 constructs and control GFP (Fig. 3c), indicating that the observed increased of WTAP in the control cells is not merely due to protein stabilization.

**Fig. 4:**
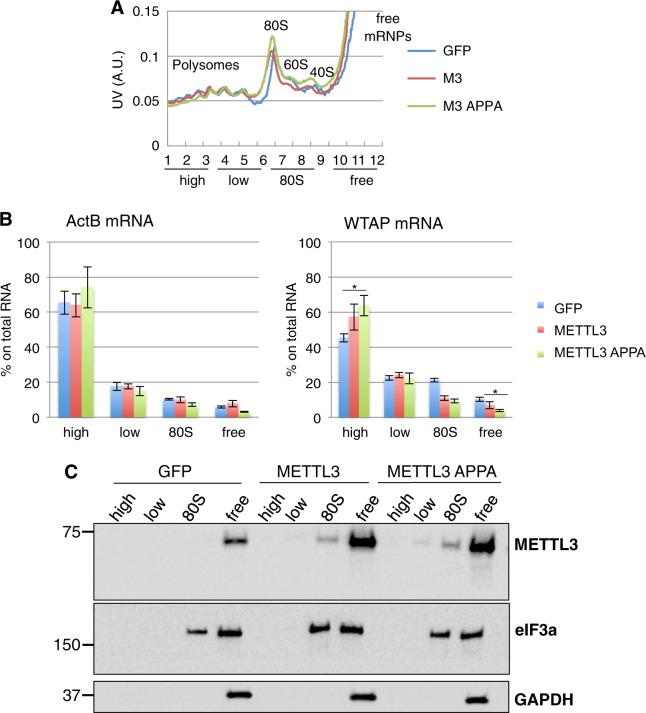
METTL3 associates with translating ribosomes and regulates WTAP mRNA translation in K562 cells. **a** Representative polysome profiles performed with K562 cytoplasmic extract. **b** ActB and WTAP mRNA distribution across the gradient was evaluated in each fraction by real-time qPCR. **c** Polysome-fractionated samples analyzed by western blot using the indicated antibodies. Data are presented as ±SEM from three independent experiments. *p < 0.05

**Fig. 5:**
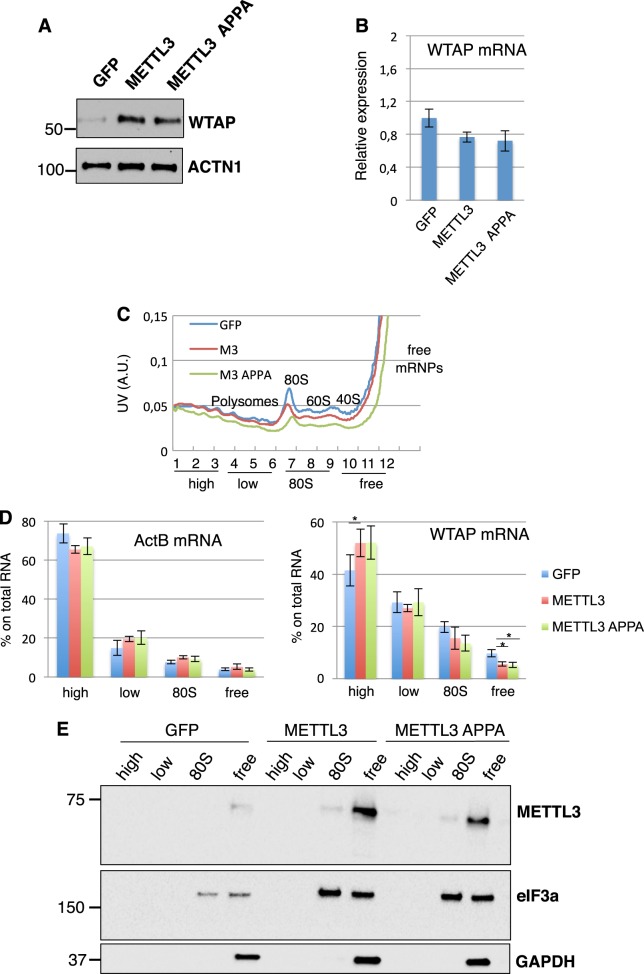
METTL3 associates with translating ribosomes and regulates WTAP mRNA translation in HeLa cells. **a** Western blot analysis of WTAP expression in HeLa cells transfected with plasmids for ectopic expression of METTL3, METTL3 APPA, and control GFP. **b** qRT-PCR analysis of WTAP mRNA in the same cells. **c** Representative polysome profiles performed on the same cells. **d** ActB and WTAP mRNA distribution across the gradient was evaluated in each fraction by real-time qPCR as described in Supplementary Methods. **e** Polysome-fractionated samples analyzed by western blot using the indicated antibodies. Data are presented as ±SEM from three independent experiments. *p < 0.05

In view of the reported activity of cytoplasmic METTL3 on mRNA translation^9^, we performed a polysome profiling by sucrose gradient centrifugation from cytoplasmic extracts prepared from control (GFP) or METTL3 (METTL3 and METTL3-APPA) stably expressing K562 cells and transiently transfected HeLa cells (Figs. 4 and 5). Similar to what we observed on K562, in HeLa cells the endogenous WTAP protein displayed an increase expression in both METTL3 and METTL3 APPA overexpression compared to GFP (Fig. 5). Again, the protein increase is not accompanied by WTAP mRNA upregulation. Then, we extracted RNA from the fractions adding an equal amount of RNA spike-in to each of them for normalization and analyzed WTAP mRNA distribution in pooled fractions representing heavy-polysomes (fractions 1–3), light-polysomes (fractions 4–6), 80S (fractions 7–9) and free-mRNAs (fractions 10–12). Then, we performed qRT-PCR on WTAP and a control mRNA, ActB (Figs. 4a,b and 5c, d). Importantly, upon overexpression of METTL3 and METTL3-APPA we observed an increase of WTAP mRNA in the polysomal fractions associated with a decrease from the free RNA fractions. Moreover, analysis by Western blot revealed that, similarly to the translation initiation factor eIF3a, METTL3 was detected with 80S and light polysomal fractions (Figs. 4c and 5e). Thus, we can conclude that METTL3 affects WTAP protein levels through translation regulation mechanisms even if, in view of the light shift into polysomal fractions, it is possible also a contribution from protein stabilization.

**Fig. 6:**
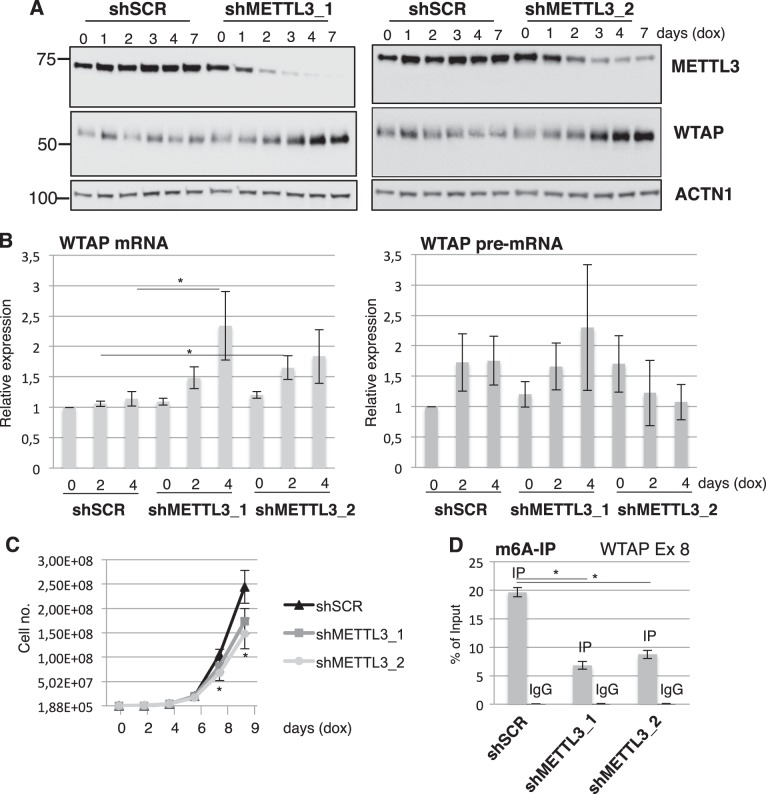
Knockdown of METTL3 results in an increase of WTAP protein levels. **a** Western blot analysis of WTAP and METTL3 expression in K562 cells infected with lentivirus expressing dox-inducible shRNAs against METTL3 (shMETTL3_1 and shMETTL3_2). A lentivirus expressing a dox-inducible scramble shRNA (shSCR) was utilized as control. **b** qRT-PCR analysis WTAP mRNA and pre-mRNA levels in the same cells. Days of dox induction are indicated. **c** Growth curve of K562 infected cells upon dox induction. **d** qRT-PCR analysis of m6A IP in K562 infected with shSCR, shMETTL3_1 or shMETTL3_2 after 6 days of dox induction. Data are presented as ±SEM from three independent experiments. *p < 0.05

In order to further investigate the relationship between METTL3 and WTAP expression, we analyzed WTAP levels upon knock down of METTL3 by using two different lentiviral vectors expressing dox-inducible shRNA in K562 cells. A non-targeting scramble shRNA was utilized as control (shSCR). Interestingly, upon METTL3 downregulation we observed an increase of both WTAP mRNA and protein levels (Fig. 6a, b). Analysis of WTAP pre-mRNA levels showed that they did not increase concomitantly with WTAP mRNA and METTL3 downregulation, indicating that the increase of WTAP mRNA is not due to transcriptional regulation (Fig. 6c). Altogether these data indicate that both up- and down- regulation of METTL3 levels result in an increase of WTAP protein and, therein, that this is not merely due to protein stabilization by METTL3 interaction. METTL3 depletion by shRNAs resulted in an inhibition of cell proliferation. However, we did not observe a significant induction of apoptosis as reported in other AML cell lines (Supplemental Figure S3). These data indicate that the increase of WTAP protein upon METTL3 knock down is not sufficient to promote cell growth. Notably, the increase of WTAP proteins preceded the arrest in proliferation and was not observed upon cell cycle arrest of K562 induced by imatinib, a tyrosine kinase inhibitor that specifically induces cell cycle arrest and apoptosis in K562 cell (Supplementary Figure S4), indicating that is specifically due to METTL3 downregulation and not to a general decrease in cell proliferation. m6A IP followed by qRT-PCR using primers to amplify the m6A peak region (exon 8) of fragmented WTAP mRNA was used to analyze the m6A methylation of WTAP mRNA in K562 AML cells. Experiments were performed 6 days after dox induction. As expected, we observed immunoprecipitation of WTAP exon 8 in control cells and a strong decrease upon METTL3 knockdown (Fig. 6d). Furthermore, the enhanced expression of WTAP mRNA coincides with the loss of m6A modifications. Therein, suggesting that the observed increase in WTAP it is dependent on the loss of m6A from its mRNA. We knocked-down the m6A reader YTHDF2, which destabilizes m6A containing mRNAs, but we did not observe an increased expression of WTAP mRNA and protein (Supplemental Figure S5). The identification of the factor(s) responsible for the modulation of WTAP expression upon METTL3 knockdown will be subject of further investigation.

**Fig. 7:**
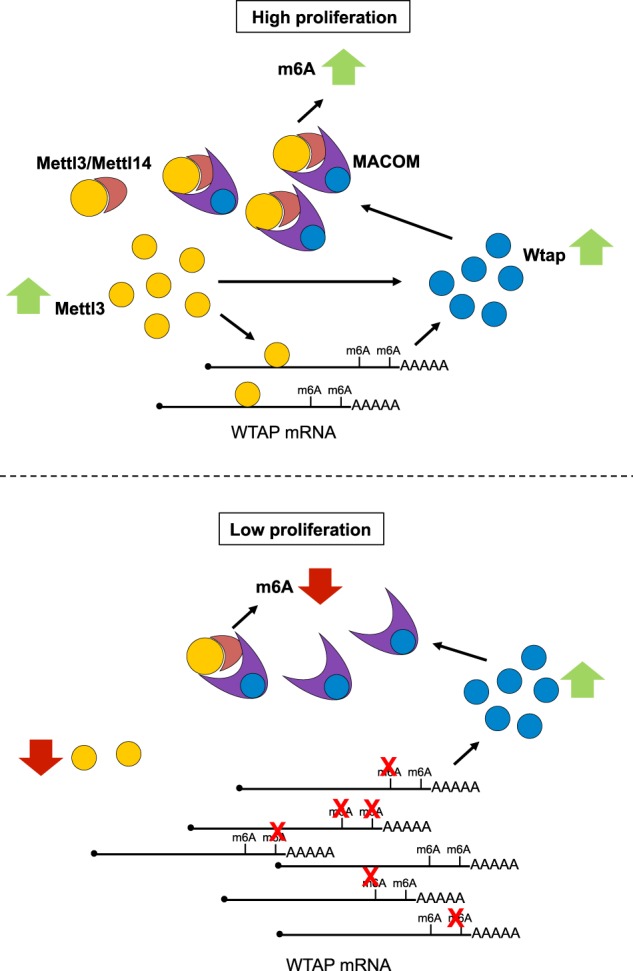
A proposed model METTL3 and WTAP interplay in AML. High levels of METTL3 produces higher WTAP protein levels by stimulating WTAP mRNA translation and WTAP protein stabilization. This results in high levels of both the MACOM complex (composed of WTAP, VIRILIZER, HAKAI, RBM15, and ZC3H13) and the METTL3/METTL14 methylation complex. This produces high m6A levels and increase cell proliferation. Conversely, depletion of METTL3 causes low activity of the METTL3/METTL14 methylation complex, which results in low m6A level in WTAP mRNA, increased WTAP mRNA stability, and WTAP protein levels. However, in the absence of the METTL3/METTL14 methylation complex, and eventually high m6A levels, this is not sufficient to promote cell growth

In conclusion, we show that METTL3 protein levels are important for WTAP protein homeostasis. In particular, we demonstrate that METTL3 can increase WTAP expression by at least two independent mechanisms (Fig. 7). First, increase of METTL3 levels can produce higher WTAP protein levels, which are independent from METTL3 catalytic activity and relies on increase WTAP mRNA translation and protein stabilization. This mechanism is relevant to increase WTAP expression concomitantly to the METTL3/METTL14 core complex and sustain the oncogenic role reported for the m6A modification complex in leukemia. Second, decrease of METTL3 levels results in increased WTAP mRNA levels and, eventually, WTAP protein. However, in the absence of a functional METTL3 the observed increase of WTAP protein is not sufficient to promote cell growth. Therein, these data indicate that the reported oncogenic function of WTAP is strictly connected to a functional m6A methylation complex.

## Materials and methods

### Cell culture and reagents

K562 cell lines were cultured at 37 °C under an atmosphere containing 5% CO_2_ in RPMI 1640 medium supplemented with 1× penicillin/streptomycin solution, 1× L-glutamine, and 10% fetal bovine serum (FBS). HeLa cells were grown in DMEM medium with 10% FBS, 1× L-glutamine, 1× penicillin–streptomycin and cultured at 37 °C under an atmosphere containing 5% CO_2_. Doxycycline (Dox), cycloheximide and Imatinib mesylate were purchased from Sigma-Aldrich.

### Plasmid constructs and cell lines

The METTL3 and WTAP DNA was amplified from K562 cDNA and cloned Hind III and Not I, and BamH I and Not I, respectively, in pcDNA3.1 vector with primers METTL3_HindIII_FW and METTL3_NotI_REV, and WTAP_BamHI_FW and WTAP_NotI_REV. METTL3 APPA mutant was obtained by reverse PCR with primers APPA_mettl3_FW and APPA_mettl3_REV. FLAG peptide sequence was introduced upstream the coding sequence of METTL3 and WTAP constructs by reverse PCR with primers METTL3_flag_FW and METTL3_flag_rev for METTL3 plasmid, and WTAP_flag_FW and WTAP_flag_rev for WTAP plasmid. FLAG WTAP Δ8 plasmid was obtained by reverse PCR on the plasmid pcDNA3.1 FLAG WTAP with primers WTAP_m6Adel_iPCR_REV and WTAP_pcDNA_FW. FLAG-METTL3 constructs were subcloned in the enhanced PiggyBac (ePB) vector ePB-PURO for stable integration^25^. This plasmid contains a TET-on system for inducible transgene expression. Helper and transposon plasmids were electroporated in K562 with Lipofectamine 2000 reagent (Invitrogen) according to manufacturer instruction. Selection with 1 μg/ml of puromycin (SIGMA) was initiated 2 days after transfection and maintained until resistant colonies became visible. Induction was obtained with dox at a concentration of 50 ng/ml. HeLa cells were transfected with pcDNA 3.1 vectors using Lipofectamine 2000 Reagent (Invitrogen) according to manufacturer instruction.

For METTL3 downregulation in K562 cells we have utilized an inducible shRNA expression system based on the lentiviral vector pLKO-Tet-On^26^. Inducible constructs were derived from Mission Lentiviral shRNA clones (Sigma-Aldrich) TRCN0000289812 (shMETTL3_1), TRCN0000289814 (shMETTL3_2) and SHC202 TRC2 (Non-Target shRNA Control) as described^26^. Selection with 1 μg/ml of puromycin (Sigma-Aldrich) was initiated 2 days after transduction and maintained until resistant colonies became visible. After selection, shRNAs induction was obtained with dox at a concentration of 50 ng/ml.

SiRNAs against YTHDF2 mRNA (Qiagen SI04174534), METTL3 (Qiagen SI04340749, SI04241265, SI04140038, SI04317096) and control siRNAs (Qiagen Negative control 1027281) were transfected in a final concentration of 30 nM using Lipofectamine RNAiMAX (Invitrogen) according to manufacturer instruction.

### Cellular mortality

Cell death was analyzed by flow cytometry (CyAN ADP DAKO) with propidium iodide (PI) exclusion assay after staining the cells with 2.5 μg/ml of PI (Sigma-Aldrich, St. Louis, MO, USA).

### Lentivirus packaging and viral transduction

Lentiviral particles were produced by calcium phosphate transient transfection of 293T cells, cotransfecting the specific lentiviral plasmid (pLKO-Tet-On shSCR, pLKO-Tet-On shMETTL3_1 and pLKO-Tet-On shMETTL3_2) together with the packaging plasmids (pLP1 and pLP2) and the envelope plasmid pLP/VSVG encoding for VSV-G protein. One 150 mm dish of 293T cells was transfected for each lentiviral construct. The calcium phosphate–DNA precipitate was allowed to stay on the cells for 14–16 h, after which the medium was replaced with complete media supplemented with 1 mM sodium butyrate (SIGMA-ALDRICH). The medium was collected 48 h after transfection, replaced with complete media supplemented with sodium butyrate 1 mM and again collected 72 h after transfection. Collected media were pulled, centrifuged at 1000 rpm for 5 min at room temperature, filtered through 0.45 μm pore nitrocellulose filters and then ultracentrifuged at 20,000 rpm for 2 h at 4 °C with SW28 rotor (Beckman Coulter). The supernatant was then removed while the pellet containing lentiviral particles was resuspended in 25 μl HBSS buffer (GIBCO ThermoFisher) and stored at −80 °C.

For viral transduction, 500.000 K562 cells were resuspended in 500 μl of serum-free and antibiotic-free media supplemented with 4 µg/ml Polybrene (SIGMA-ALDRICH). Cells were then infected with 5 μl of the lentiviral particles previously resuspended in HBSS buffer. After 6 h, one volume of medium with serum 2× and antibiotic 2× was added. 24 h after viral transduction the medium was replaced with complete medium. 48 h after viral transduction cells were selected with 1.5 µg/ml of puromycin (SIGMA-ALDRICH) until resistant colonies became visible (3–5 days).

### Protein stability assay

After 48 h from transient transfection, Hela cells were treated with 100 μg/ml of cycloheximide for 4 h. We used non-treated cells as control cells. Cells were than collected and protein fraction was analyzed. Notably, we analyzed protein levels by western blot using equal volumes of different samples.

### RNA extraction and real-time qRT-PCR analysis

Total RNA was extracted using the Quick RNA miniprep kit (Zymo) according to manufacturer instructions. For mRNA analysis, reverse transcription to cDNA was performed with the SuperScript VILO cDNA Synthesis Kit (Life Technologies) according to the manufacturer instructions. Quantitative real-time PCR was performed on an Applied Biosystems 7500 Fast Real Time PCR System. Reactions were performed in triplicate using the SYBR green dye detection system and analyzed using 7500 Software v2.0.6 (Applied Biosystems). Relative expression levels of targets were determined using the comparative 2∆∆Ct method. ActB mRNA was utilized as a reference (primers: ACTB_hs_FW, ACTB_hs_REV). METTL3 was analyzed with oligos METTL3 SYBR FW and METTL3 SYBR REV, WTAP with WTAP SYBR FW and WTAP SYBR REV, FLAG WTAP with FLAG_FW and WTAP SYBR REV2, endogenous WTAP with WTAP_5′UTR_FW and WTAP SYBR REV2, endogenous WTAP with WTAP_5′UTR_FW and WTAP SYBR REV2, WTAP long isoform with WTAP_long_FW and WTAP_long_REV, WTAP pre-mRNA with WTAP pre-mRNA FW and WTAP pre-mRNA REV, HPRT with HPRT SYBR FW and HPRT SYBR REV, YTHDF2 with YTHDF2 SYBR FW and YTHDF2 SYBR REV, TAZ with TAZ SYBR FW and TAZ SYBR REV.

### Nuclear/cytoplasmic fractionation

For nucleus to cytoplasm separation approximately 10 × 10^6^ cells were pelleted and washed with PBS without calcium and magnesium, then they were resuspended in 100 μl of Buffer A (200 mM Tris HCl pH 8, 10 mM NaCl, 3 mM MgCl_2_, 0.1% NP40, 10% glycerol, 0.2 mM EDTA, 1 mM DTT) complemented with PIC 1× (Complete, EDTA free, Roche). Cells were incubated on ice in Buffer A for 10′, and then centrifuged at 2000 rpm for 5′ at 4 °C. The supernatant contains the cytoplasmic extract. Buffer A is added to the pellet containing the nuclei and the resuspended pellet is centrifuged again for washing. After washing, the pellet is resuspended in Buffer C (20 mM Tris HCl pH 8, 400 mM NaCl, 20% glycerol, 1 mM DTT) complemented with PIC 1× (Complete, EDTA free, Roche). The nuclei are subjected to thermal shock with three cycles of freezing in liquid nitrogen and thawing at 37 °C. After thermal shock the extract is centrifuged at 13,000 rpm for 15′ at 4 °C. The supernatant contains the nuclear extract.

### Immunoblot analysis

30 μg of whole cell extract was separated by 10% SDS-PAGE and electroblotted to nitrocellulose membrane (Protran, S&S). Immunoblots were incubated with antibodies Anti-FLAG M2 F3165 (Sigma-Aldrich), Anti-WTAP 60188-1-Ig (Proteintech), Anti-METTL3 [EPR18810] (Abcam), Anti-METTL14 antibody HPA038002 (Sigma Aldrich), anti-Actinin H-300 sc-15335 (Santa Cruz Biotechnology), anti-GAPDH sc-25778 (Santa Cruz Biotechnology), anti-YTHDF2 NBP2-31785 (Novus Biological) and anti-eIF3A ab86146 (Abcam).

### m6A immunoprecipitation

m6A immunoprecipitation was performed as described in ref. ^27^ with a few modifications. Briefly, K562 cells infected with lentiviral vectors expressing dox-inducible shRNAs shSCR, shMETTL3_1 and shMETTL3_2 were induced with dox 50 ng/ml. 6 days after induction total RNA was extracted and fragmented into ~100 nt long fragments in Fragmentation Buffer (100 mM Tris–HCl and 100 mM ZnCl_2_) for 5′ at 94 °C. Reaction was immediately blocked with addition of EDTA 50 mM. A portion of fragmented RNA was kept as input control, while 50 μg of fragmented RNA were immunoprecipitated in 1 ml of IP Buffer (50 mM Tris–HCl, 750 mM NaCl and 0.5% Igepal CA-630) complemented with RNasin (400 U), with 2 μg of m6A-specific antibody (ab151230, Abcam) or 2 μg of control rabbit IgG (Millipore) for 2 h of incubation at 4 °C on rotator. Then 20 μl of protein A beads (Invitrogen), saturated with BSA (SIGMA) 0.5 μg/ml for 2 h, were added and the reaction mixtures and incubated for 2 h at 4°C on rotator. After incubation beads were spinned down and washed three times with IP Buffer. Elution was performed incubating the beads four times in Elution Buffer (150 mM NaCl, 50 mM Tris–HCl pH 7.5, 1 mM EDTA, 0.1% SDS, 20 mM DTT) for 5′ at 42 °C. Eluted RNA was precipitated with addition of one-tenth volumes of 3 M sodium acetate (pH 5.2), and 2.5 volumes of 100% ethanol and incubated overnight at −80 °C. Precipitated RNA was then centrifuged at 15,000*g* for 25′ at 4 °C and pellet resuspended in 15 μl of RNase-free water. qRT-PCR on immunoprecipitated RNA was performed with primers WTAP_long_FW and WTAP_long_REV.

### Cross-linking immunoprecipitation

K562 cells stably expressing FLAG-METTL3 or FLAG-METTL3 APPA were induced with dox 50 ng/ml. 48 h after induction, cells were cross-linked in PBS at 1500 × 100 μJ/cm^2^. Cells were pelleted and washed with PBS without Calcium and Magnesium, then they were resuspended in 1 ml of Buffer A (200 mM Tris HCl pH 8, 10 mM NaCl, 3 mM MgCl_2_, 0.1% NP40, 10% glycerol, 0.2 mM EDTA, 1 mM DTT) complemented with PIC 1× (Complete, EDTA free, Roche) and RNase Inhibitor (Invitrogen). Cell lysate was incubated on ice for 5′ and then centrifuged at 2000 rpm for 5′ at 4 °C, the supernatant contains the cytoplasmic extract. The cytoplasmic extract was brought to a higher molarity with an equal RIPA buffer 2× (NaCl 190 mM, NP40 0.9%, EDTA 0.8 mM) complemented with DTT 1 mM, PIC 1× and RNase Inhibitor 1×. 30 μl of protein G beads (Invitrogen) were washed twice with PBS-T buffer (PBS, TWEEN 0.02%) and then incubated with 7 μg of Anti-FLAG M2 F3165 (Sigma-Aldrich) antibody or 7 μg of mouse IgG as negative control at room temperature for 1 h. Subsequently, the beads were washed twice with PBS-T buffer and incubated at 4 °C on rotator overnight with 1.5 mg of cellular cytoplasmic extract freshly prepared. Beads were then washed with HIGH SALT WASH buffer three times (PBS 10× SIGMA diluted to have a final concentration of 500 mM NaCl, NP-40 0.5%) complemented with PIC 1× and RNase Inhibitor.

Finally, the immunoprecipitated extract was split for protein and RNA analysis. 50 μl were denatured in Laemmli Sample Buffer (Bio-Rad) and DTT 50 mM for protein analysis by Western Blot. The RNA fraction (150 μl) was subjected to reversion of crosslinking with Proteinase K 4 mg/ml at 50 °C for 30′. Afterwards, RNA was isolated for qRT-PCR analysis and normalized on a spike-in RNA (mouse long non-coding transcript).

CLIP experiments in HeLa cells were performed using the same protocol 48 h after transient co-transfection with pcDNA3.1 METTL3 or pcDNA3.1 METTL3 APPA together with pcDNA3.1 FLAG WTAP Δ8. In this case, we utilized protein A beads (Invitrogen) and 4 μg of Anti-METTL3 [EPR18810] (Abcam) antibody or 4 μg rabbit IgG as negative control (Millipore).

### Polysome profiling

Cytoplasm fractionations on sucrose gradients were performed as follows: 20 × 10^6^ cells were lysed with 500 μl of lysis buffer (10 mM Tris pH 7.5, 100 mM NaCl, 10 mM MgCl_2_, 0.5% Triton X-100, and 0.5% sodium deoxycholate) supplemented with 100 mg/ml cycloheximide, 1× PIC (Complete, EDTA free, Roche) and 1× RNase guard (Thermo Scientific). The lysates were centrifuged for 5 min at 2000 rpm at 4 °C. The supernatants were collected and centrifuged on 15–50% sucrose gradient at 37,000 rpm with a SW41 rotor (Beckman) for 2 h at 4 °C. Fractions were collected with a Bio-logic LP (Biorad). 35 μl of each fraction were pooled together 3 by 3 obtaining four fractions (Heavy Polysomes, Light Polysomes, 80 S, Free RNA). 900 μl of Qiazol (Qiagen) was added to each 100 μl fraction and 1 pg of spike in RNA (mouse long non-coding transcript) was added to each extraction for further normalization. RNA was extracted using RNeasy Mini Kit (Qiagen) according to manufacturer instruction.

### Data and statistical analysis

Microarray data were downloaded from The Cancer Genome Atlas (TCGA): TCGA_LAML dataset; and Gene Expression Omnibus (GEO): GSE12662, GSE19429, GSE12662, GSE16020, GSE16837, GSE37416, GSE42519, GSE55849, GSE72642, GSE6054, GSE13899, GSE16836, GSE60601, GSE66936, GSE72642, and GSE76803 datasets. Downloaded data were obtained from the GeneChip Human Genome U133 Plus 2.0 Array (GPL570) platform and have been normalized with the DNA-Chip Analyzer (dChip) software^28^. The normalization was performed using an array with median overall intensity chosen as the baseline array against which other arrays are normalized at probe intensity level. In this way the brightness of the arrays was adjusted to comparable level. We utilized the same software to compute model-based expression values for each array. LIMMA package was utilized for statistical analysis of differential expression^29^. Different p values obtained for each probe of the same gene were combined using the Fisher test. *p* < 0.001 calculated on AML and CD34 with respect to mature myeloid cells was considered as statistically significant. MeRIP-Seq (GSM2010455, GSM2010456), m6A CLIP (GSM1828594, GSM1828596, GSM2300426) and METTL3 PAR-CLIP (GSM1135006, GSM1135007) datasets were obtained from GEO.

Data from real time PCR analysis were subjected to the two-tailed Student’s *t* test. All values in figures are presented as the mean ± standard error of mean (SEM) of *n* independent experiments. *p* Values of <0.05 were considered to be statistically significant and indicated by 1 asterisk in figures.

### Oligonucleotides

Cloning:

METTL3_HindIII_FW: CKnockdown of METTL3 results in an increase of WTAP protein levelsGGACACGTGGAGC

METTL3_NotI_REV: ATTTGCGGCCGCCTATAAATTCTTAGGTTTAGAGAT

WTAP_BamHI_FW: CGCGGATCCATGACCAACGAAGAACCTCT

WTAP_NotI_REV: ATTTGCGGCCGCTTACAAAACTGAACCCTGTACA

APPA_mettl3_FW: CCCGCCGATATTCACATGGAACTGCCCTAT

APPA_mettl3_REV: TGGGGCAGCCATCACAACTGCAAACT

WTAP_flag_FW: GACGACGATAAGACCAACGAAGAACCTCTTCCCAA

WTAP_flag_FW: GACGACGATAAGACCAACGAAGAACCTCTTCCCAA

WTAP_flag_REV: ATCCTTGTAATCCATGGATCCGAGCTCGGTACCAA

METTL3_flag_FW: GACGACGATAAGTCGGACACGTGGAGCTCTATCC

METTL3_flag_rev: ATCCTTGTAATCCATAAGCTTAAGTTTAAACGCTAGCCA

WTAP_m6Adel_iPCR_REV: TTCATCCTGACTGCTTTTAAGCTC

WTAP_pcDNA_FW: TAAGCGGCCGCTCGAGT

qRT-PCR:

ACTB_hs_FW: CGTACCACTGGCATC

ACTB_hs_REV: GTAGTCAGTCAGGTCCCGGC

METTL3 SYBR FW: AAGCAGCTGGACTCTCTGCG

METTL3 SYBR REV: GCACTGGGCTGTCACTACGG

WTAP SYBR FW: TGCGACTAGCAACCAAGGAA

WTAP SYBR REV: ATCTCAGTTGGGCAACGCTC

WTAP SYBR REV2: CTGTGTACTTGCCCTCCAAAG

WTAP_pre-mRNA FW: TCATTTTGTGATGGATGGCTCT

WTAP_pre-mRNA REV: TCAAGTTGTGCAATACGTCCC

WTAP_5′UTR_FW: TTCTGCCTGGAGAGGATTCA

WTAP_long_FW: TCCAGTCATGACCCTCAAGAG

WTAP_long_REV: AGTCCAAGCCATTCTGAACG

HPRT SYBR FW: GCCATCACATTGTAGCCCTCTG

HPRT SYBR REV: TTTATGTCCCCTGTTGACTGGTC

FLAG_FW: GATTACAAGGATGACGACGATAAG

YTHDF2_SYBR_FW: GAACCTTACTTGAGTCCACAG

YTHDF2_SYBR_REV: GTAGGGCATGGCTGTGTCAC

TAZ_SYBR_FW: TCACTGTGCTGATCGGGAAG

TAZ_SYBR_REV: TCTCCACAGCCGACTTGTTC

## Electronic supplementary material

Supplemental Figure 1

Supplemental Figure 2

Supplemental Figure 3

Supplemental Figure 4

Supplemental Figure 5
