# Supplementary material for: METTL3 regulates WTAP protein homeostasis
Source: Cell Death Dis. 2018 Jul 23;9(8):796. doi: 10.1038/s41419-018-0843-z (PMC6056540; doi:10.1038/s41419-018-0843-z)
Supplement: Supplementary file 1 — Supplemental Figure 1 [file 41419_2018_843_MOESM1_ESM.pdf]

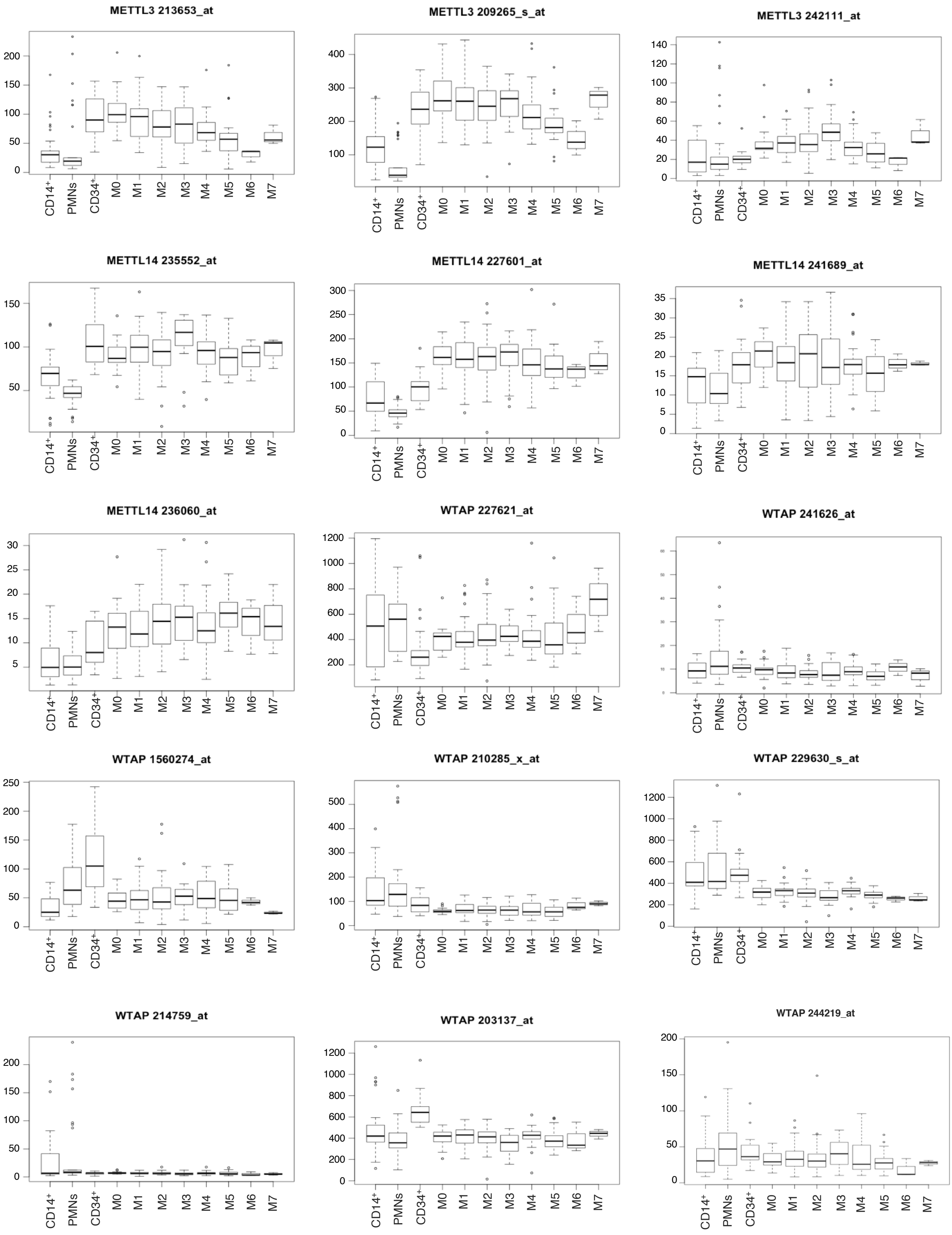

Figure S1. METTL3, METTL14 and WTAP expression in AML of different FAB subtypes, M0 (n=15), M1 (n=43), M2 (n=42), M3 (n=17), M4 (n=36), M5 (n=22), M6 (n=3) and M7 (n=3); normal CD34+ hematopoietic progenitors (n=22) and mature myeloid cells, normal CD14+ monocytes (n=34) and polymorphonuclear leukocytes (PMNs, n=30). Data were obtained from public microarray repositories (see Supplemental Materials). The box plots illustrate the distribution of expression values of all probes present in the microarray for indicated gene; the central solid line indicates the median; the limits of the box show the upper and lower percentiles.
