# Supplementary material for: METTL3 regulates WTAP protein homeostasis
Source: Cell Death Dis. 2018 Jul 23;9(8):796. doi: 10.1038/s41419-018-0843-z (PMC6056540; doi:10.1038/s41419-018-0843-z)
Supplement: Supplementary file 2 — Supplemental Figure 2 [file 41419_2018_843_MOESM2_ESM.pdf]

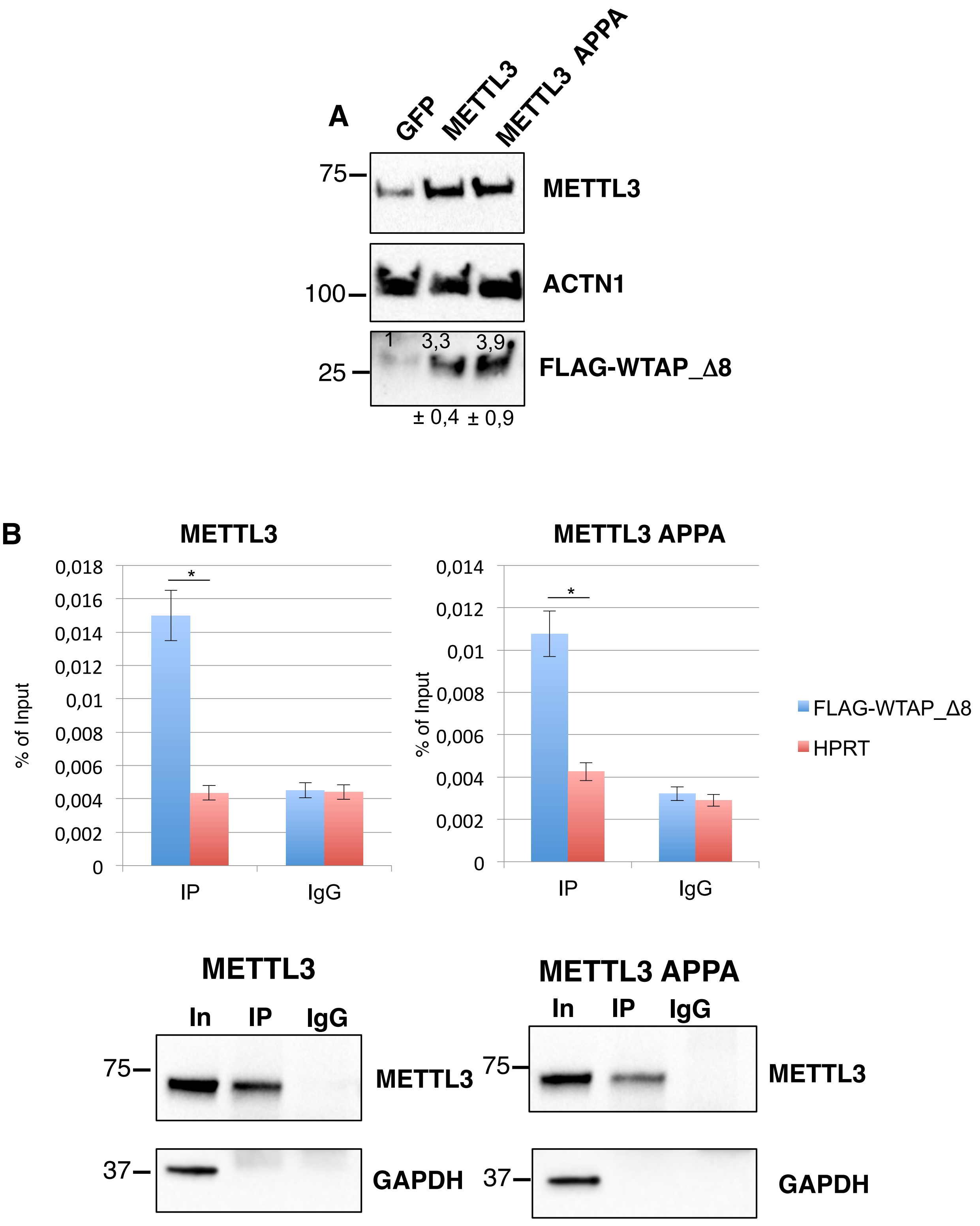

Figure S2.(A) Western blot analysis of HeLa cells transfected with a plasmid for transient expression of FLAG-WTAP_Δ8 together with plasmids expressing a GFP control, METTL3 or METTL3 APPA. (B) Upper panel, qRT-PCR of CLIP experiments performed in the same cells of (A). Lower panel, representative Western blot analysis. Data are presented as ± S.E.M from three independent experiments. P < 0,05 = * .
