# Supplementary material for: METTL3 regulates WTAP protein homeostasis
Source: Cell Death Dis. 2018 Jul 23;9(8):796. doi: 10.1038/s41419-018-0843-z (PMC6056540; doi:10.1038/s41419-018-0843-z)
Supplement: Supplementary file 3 — Supplemental Figure 3 [file 41419_2018_843_MOESM3_ESM.pdf]

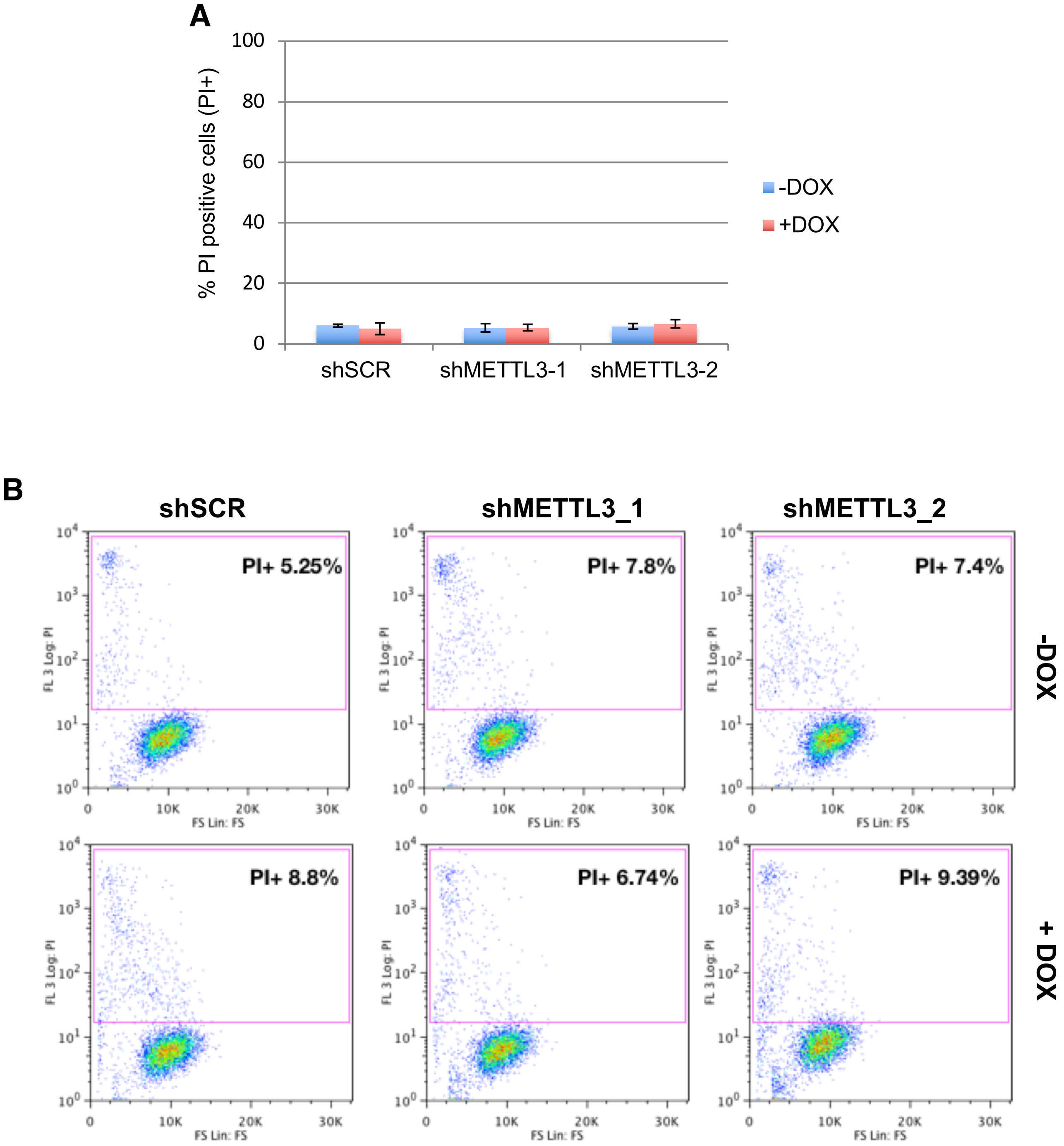

Figure S3.(A) The histogram represents the percentage of propidium iodide (PI) positive K562 cells trasduced with shSCR, shMETTL3_1 and shMETTL3_2 and treated for 6 days with doxycycline (+DOX). Untreated cells (-DOX) were utilized as control. Data are presented as ± SD from three independent experiments. (B) Representative FACS analysis PI positive cells.
