# Supplementary material for: METTL3 regulates WTAP protein homeostasis
Source: Cell Death Dis. 2018 Jul 23;9(8):796. doi: 10.1038/s41419-018-0843-z (PMC6056540; doi:10.1038/s41419-018-0843-z)
Supplement: Supplementary file 4 — Supplemental Figure 4 [file 41419_2018_843_MOESM4_ESM.pdf]

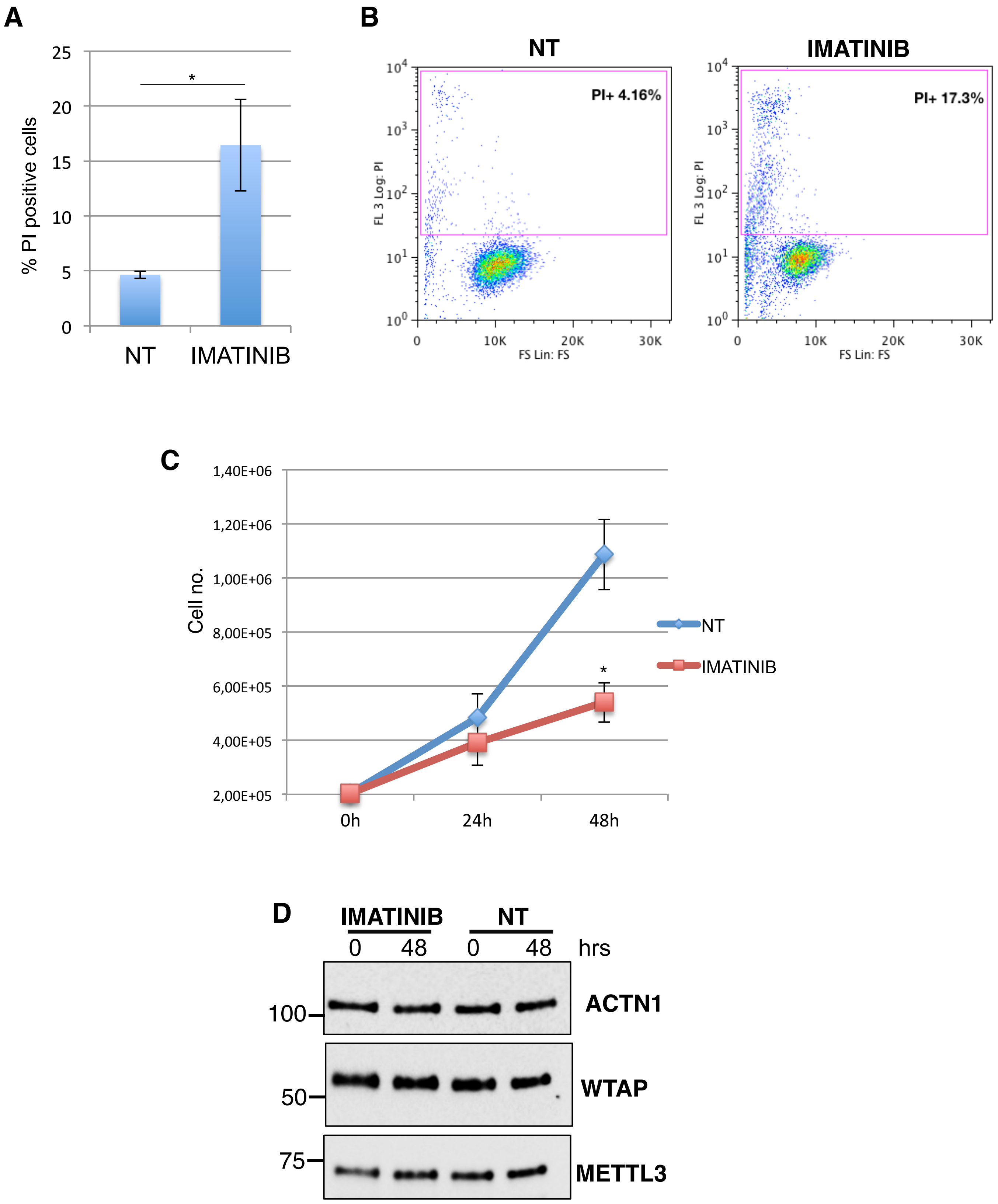

Figure S4.(A) The histogram represents the percentage of propidium iodide (PI) positive K562 cells treated for 48 hours with imatinib (250 nM). Untreated cells (NT) were utilized as control. (B) Representative FACS analysis. (C) Growth curve of K562 cells treated (IMANITIB) or non treated (NT) with imanitib. (D) Representative Western blot analyis of K562 cells treated or non treated with imatinib (250 nM). Expression differences were statistically analysed with Student's t test; p < 0,05 = * .
