# Supplementary material for: METTL3 regulates WTAP protein homeostasis
Source: Cell Death Dis. 2018 Jul 23;9(8):796. doi: 10.1038/s41419-018-0843-z (PMC6056540; doi:10.1038/s41419-018-0843-z)
Supplement: Supplementary file 5 — Supplemental Figure 5 [file 41419_2018_843_MOESM5_ESM.pdf]

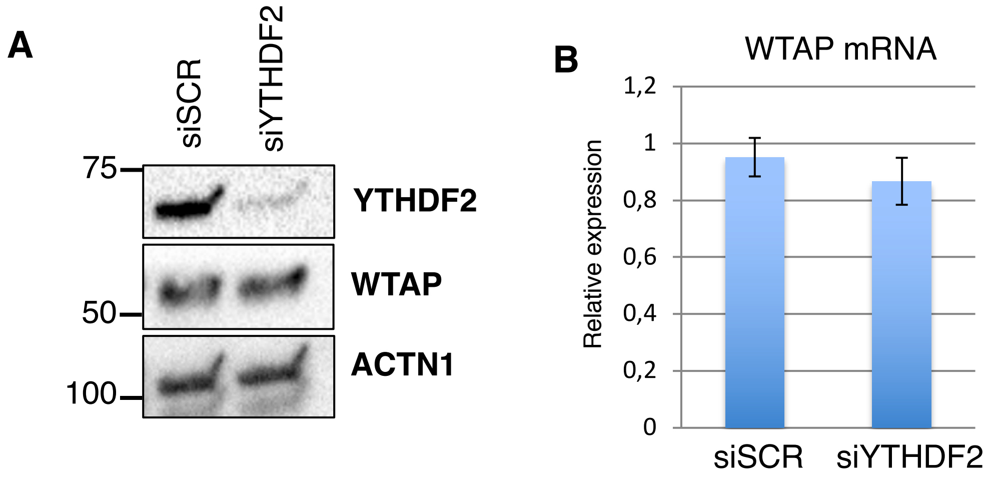

Figure S5.(A) Representative Western blot analysis of WTAP expression in HeLa cells transfected with siSCR or siYTHDF2. (B) qRT-PCR analysis of WTAP mRNA in the same cells. Experiments were performed in triplicate. Data are presented as ± SD from three independent experiments.
